# Dr. Yeats's Reply to Mr. Gibbon

**Published:** 1813-09

**Authors:** G. D. Yeats

**Affiliations:** Bedford


*To the Editors of the Medical and Physical Journal.*

GENTLEMEN,

THE difference of opinion which originally took place on the disputed case, arose entirely between Mr. Gibbon and myself on account of the symptom of vomiting of urine. I could not agree to the opinion that such a symptom was totally impossible. This circumstance rendered the case notorious. The *onus probandi* then lay with me, which induced me to say, in the conversations I had with individuals, that I would publish and prove it. The paper on Ischuria contains the reasons and proofs of my opinion, and I am not the only one who considers the facts adduced in that paper as placing the occurrence of the disputed symptom, even for a considerable length of time, beyond the possibility of a doubt, as the Q. E. D. and the redemption of any pledge I may have given on this point. My feeling has been all along absorbed in the consideration of this subject, and but for the denial of the utter possibility of the symptom, I should not have engaged in the discussion at all. The reasons for publishing the paper in the form in which it appears are before the public. Having thus published according to what have been my feeling and intention as to the immediate redemption of any pledge given, the *onus refutandi* now rests with any gentleman who thinks proper to dispute the point; but an explanatory answer, if it appeared necessary, would be more immediately due from me to Mr. Gibbon, as between us the difference of opinion originally commenced.

That I have said I would publish the case, and that that expression was uttered with an intention on my part so to do, I do not at all dispute; but, however forcibly or loosely such expressions came from me, the times and seasons for such a production are certainly to be chosen by me. Not feeling myself under any public pledge properly so called, more especially after the paper on ischuria, adducing proofs of vomiting of urine, the original and only matter of difference notoriously between Mr. Gibbon and myself, I cannot acknowledge the right to demand that which a willing endeavour would be made to grant, to the wishes of Mr. Pulley or any other gentleman whatsoever.

Nothing that I have personally witnessed, or that has been stated to me by the patient herself, conveys to my mind the conviction of imposition. If, however, facts are known clearly proving that the patient has practised the arts of imposture, let all these circumstances be communicated, for it must not be forgotten that I was not present at the meeting of the gentlemen who took these circumstances into consideration, as mentioned in my last communication. In any stage of an inquiry, unquestionably I would readily yield my assent that imposition was practised, if circumstances had sufficient weight with me to produce that conviction; but if that opinion is to be maintained solely on the ground that vomiting of urine, even for a considerable time, is utterly impossible, I have stated the facts from which I must continue an infidel on what was the original question, and which was the only point I ever considered myself strictly bound to prove.

*Bedford, August* 8, 1813. G. D. YEATS.

